# Emergency Department Quality Dashboard; a Systematic Review of Performance Indicators, Functionalities, and Challenges

**DOI:** 10.22037/aaem.v9i1.1230

**Published:** 2021-06-17

**Authors:** Sohrab Almasi, Reza Rabiei, Hamid Moghaddasi, Mojtaba Vahidi-Asl

**Affiliations:** 1 Department of Health Information Technology and Management, School of Allied Medical Sciences, Shahid Beheshti University of Medical Sciences, Tehran, Iran.; 2 Faculty of Computer Science and Engineering, Shahid Beheshti University, Tehran, Iran.

**Keywords:** Emergency service, hospital, quality indicators, health care, data management, systematic review

## Abstract

**Introduction:**

Effective information management in the emergency department (ED) can improve the control and management of ED processes. Dashboards, known as data management tools, efficiently provide information and contribute greatly to control and management of ED. This study aimed to identify performance indicators quality dashboard functionalities, and analyze the challenges associated with dashboard implementation in the ED.

**Methods:**

This systematic review began with a search in four databases (Web of Science, PubMed, Embase, and Scopus) from 2000 to May 30, 2020, when the final search for papers was conducted. The data were collected using a data extraction form and the contents of the extracted papers were analyzed through ED performance indicators, dashboard functionalities, and implementation challenges.

**Results:**

Performance indicators reported in the reviewed papers were classified as the quality of care, patient flow, timeliness, costs, and resources. The main dashboard functionalities noted in the papers included reporting, customization, alert creation, resource management, and real-time information display. The dashboard implementation challenges included data sources, data quality, integration with other systems, adaptability of dashboard functionalities to user needs, and selection of appropriate performance indicators.

**Conclusions:**

Quality dashboards facilitate processes, communication, and situation awareness in the ED; hence, they can improve care provision in this department. To enhance the effectiveness and efficiency of ED dashboards, officials should set performance indicators and consider the conformity of dashboard functionalities with user needs. They should also integrate dashboards with other relevant systems at the departmental and hospital levels.

## 1. Introduction:

Since the emergency department (ED) provides complex services and hosts patients in critical physical conditions, timely and precise service provision in ED is considered a challenge(1). Moreover, the increased number of visits to the ED disrupts emergency care provision in this department(2, 3). These problems lead to negative outcomes for both patients (morbidity and mortality) and healthcare providers in the ED (stress and burnout)(4). Effective information management in the ED provides timely information and improves process control and management in this department (5). The use of information technology in the ED plays a pivotal role in information management and enhances managerial and treatment-related processes(6). However, such systems are either not used at all or designed in a way that they further increase the workload of healthcare providers in the ED(7). Research shows that although these systems promote coordination and communication among healthcare providers, they fail to offer rapid access to patient information, delay information recording, and have poor user-friendliness.(8) A poor user interface design is another disadvantage of ED information systems, which can cause users to face problems in accessing the necessary information (9, 10). Dashboards, known as data management tools in the ED, collect data from various information systems including the ED, laboratory, and radiology information systems and display them based on pre-defined key performance indicators. Using dashboards, ED managers evaluate the performance of their department, identify problems, analyze their causes, and thus promote their own performance(11). By displaying information through visual tools, dashboards help managers visually identify trends and patterns. The use of quality dashboards in the ED also facilitates work processes, reduces hospitalization duration, better demonstrates the ED status, improves coordination, enables rapid access to information, decreases complications for patients, promotes the monitoring of performance indicators by managers, enhances reporting flexibility, and provides timely information (12, 13). The key to a suitable design is paying attention to functionalities and performance indicators monitored by dashboards. Dashboard functionalities demonstrate the system operations or the activities performed/facilitated by these operations (12). Classified as a type of dashboard, the quality dashboard collects information from various sources and, by using visual tools based on key performance indicators, provides information at the level of department or organization to help users with decision-making (12-14). Quality dashboards include those of the operating room (15), radiology (16), nursing (17), and intensive care unit (18). The use of dashboards enhances management of department processes, improves communications, and thus, aids decision-making. 

It is important to employ quality dashboards to obtain timely information for effective control and management of ED processes. For effective information management and communications in the ED, key performance indicators and functionalities of the quality dashboards should also be determined in terms of user needs (12, 19). This study aimed to identify ED performance indicators and quality dashboard functionalities, and analyze the challenges associated with their implementation in the ED.

## 2. Method

***2.1. Data sources and search strategy***

Data search and extraction phases were performed based on the Preferred Reporting Items for Systematic Reviews and Meta-Analyses (PRISMA) checklist (20). The search formula was adopted through a combination of MeSH terms, Emtree, and keywords pertaining to dashboards and the ED (Table 1). The search for finding relevant papers was conducted in four databases (Embase, Web of Science, PubMed, and Scopus) (Table 2). 

The final search for papers was conducted on May 30, 2020. Moreover, this study spanned from June 1 to December 30. One researcher (SA) independently searched and retrieved the papers, whereas uncertainties were discussed with the other two authors (RR and HM). The relevant papers were also retrieved through a search in Google Scholar and Google. The search for papers was finalized with a bibliographic check of the designated papers. 

***2.2. Inclusion and exclusion criteria***

The inclusion criteria were: 1) papers written in English; 2) papers on ED quality dashboards; and 3) papers on performance indicators and functionalities of ED quality dashboards. Moreover, the exclusion criteria were: 1) non-English papers; 2) papers merely designing a clinical dashboard for the ED; or 3) papers examining the dashboards of other hospital wards.

***2.3*** **.** ***Paper selection, paper evaluation, and data extraction***

In the screening step, three authors (SA, RR, and HM) checked the titles and abstracts of the papers and irrelevant papers were removed. In the eligibility step, the papers were independently checked by the same noted authors. The bibliography check was then conducted by one of the authors (SA). The quality assessment of the papers based on Cochrane Effective Practice and Organization of Care (EPOC) guideline (21) was independently conducted by SA, RR, and HM.

For data extraction, the performance indicators of the ED were first extracted from the papers. The performance indicators used by the ED quality dashboards mentioned in the reviewed papers were classified as patient flow, timeliness, quality of care, costs, and resources (Table 3) (22). The first author’s name, the year of publication, the place of study, the quality dashboard functionalities, and the main challenges associated with using the dashboard in the ED were then extracted from each paper (Table 4). 

## 3. Results:

**Figure 1:**
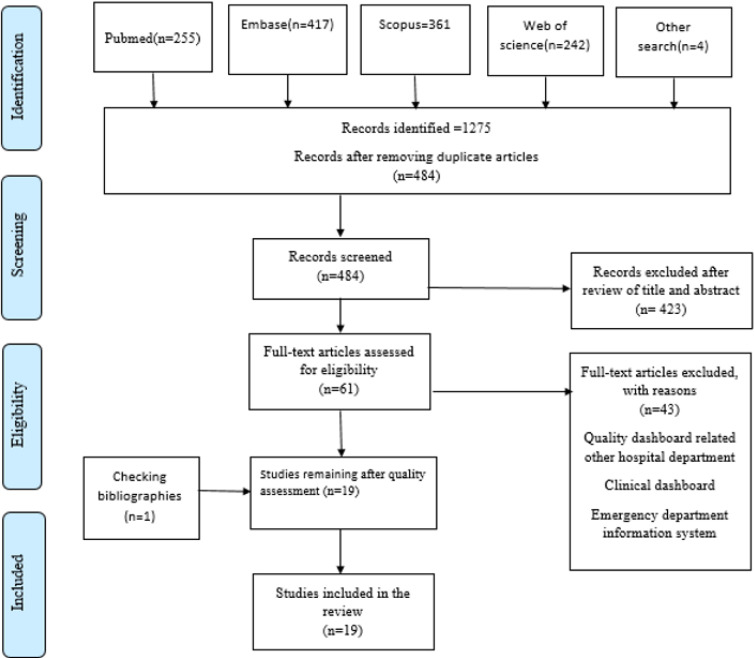
Flow diagram based on PRISMA

A total of 1275 papers were retrieved through the search in databases. Additionally, four papers were retrieved from a search attempt on Google Scholar and Google. After removing the duplicates using endnote software, 484 papers remained. The titles and abstracts of the papers were then reviewed. As a result, 423 papers were removed and 61 remained. In the next step, the full texts of papers were checked and this resulted in the removal of 42 papers. Ultimately, 18 papers remained for analysis. One more paper was also retrieved when performing a bibliographic check of the designated papers. No paper was removed after quality assessment, and all the papers entered the final analysis phase. Finally, 19 papers were reviewed in this study. Figure 1 displays the paper selection process.

***3.1.*** ***Quality assessment***

According to the quality assessment of papers, three studies (19, 23, 24) were considered as “high quality”; four studies(25-28) were introduced as “fair to good quality”, and 12 studies(11, 29-39) were regarded as low quality (Table 5). 

***3.2. ED quality dashboard performance indicators***

The performance indicators (26 in total) were reported in five main categories (quality of care, patient flow, timeliness, costs, and resources). The majority of performance indicators were related to patient flow (8 indicators), timeliness (7 indicators), quality of care (4 indicators), and costs (2 indicators), respectively. The following indicators belonged to the patient flow category: the number of patients discharged and the type of discharge (referral to other centers, admission at the hospital) (in 11 papers), the total number of people visiting the ED (n=10), the number of patients in the ED (divided by age, gender, type of specialty, and triage level) (n=8), the number of patients admitted per triage level (n=6), how the patients visited the ED (personal vehicles, ambulance, on foot), and the number of patients for whom a decision was made in six hours (n=4). The timeliness category consisted of the following performance indicators: the patients’ mean length of stay (n=16), the time of triaging (per each triage level) (n=4), and the mean time elapsed since the patients’ arrival in the ED until the onset of triaging (n=4). Only three out of the 19 papers had mentioned the quality of care performance indicators, which included patient revisits (before 72 hours) (n=3) and the percentage of mortality among the patients admitted to the ED (in=2). The cost performance indicators included the number of tests ordered by the doctors (n=8) and the number of consultations given (n=3). Finally, resource performance indicators were the number of beds (available, extra, occupied, reserved, and out-of-order) (n=7) and the number of personnel in the ED (divided by discipline and gender) (n=6).

***3.3. ED quality dashboard functionalities***

All the 19 papers discussed the reporting functionality. This was followed by customization (n=12)(11, 19, 23, 25-27, 29-32, 34), alerting (n=10)(11, 24, 26, 27, 30, 32-35), resource management (n=9)(19, 25, 26, 29-32), and real-time information display (n=8)(11, 19, 24, 25, 27, 28, 38, 39) as the most frequently mentioned functionalities, respectively. Other papers also reported functionalities such as automated data collection (n=4) (11, 19, 27, 38) and the use of drill-up, drill-down, and drill-through (n=4)(23, 24, 26, 27).

***3.4. Challenges to use of quality dashboards in ED***

The challenges associated with the use of quality dashboards in the ED included a lack of integration with other hospital systems and inputting the data manually(25, 32), lack of adaptability to work processes in the ED and lack of flexibility (24, 28, 29, 31, 33-35), breaching patient confidentiality by displaying patients’ names and test results on the dashboard’s large screen(27, 30), a problem with understanding and interpreting the type of information displayed through visualization tools (30, 31), and the accuracy of the data entered into the dashboard (23).

## 4. Discussion:

This study aimed to identify the performance indicators, functionalities, and challenges of quality dashboards in the ED. Some studies had examined the effects of clinical/quality dashboards on patient care improvement or analyzed the functionalities and positive and negative effects of using dashboards in hospital(40, 41). Determining key performance indicators lays the ground for performance measurement, ensures progress evaluation based on pre-defined goals/criteria, and aids managers in decision-making by providing timely and appropriate information (42). Based on the results of this study, ED performance indicators are divided into five groups of quality of care, patient flow, timeliness, costs, and resources. These findings are in line with the results of studies by Sørup (22) and the US Institute of Medicine (43). Since the patient-centeredness indicator deals with the provision of care based on patients’ needs, preferences, and values, this indicator was called patient flow in this study (43). Safety deals with the perceived damage and complications of treatment processes. Since the patient mortality indicator may not be related to treatment processes at the hospital and result from underlying diseases (44, 45), this indicator was replaced by the quality of care in this study. As for the quality dashboard functionalities, our findings yielded reporting, customization, real-time information display, resource management, and alerts. Previous studies have introduced measurement, monitoring, collection, processing, performance measurement, and reporting as dashboard functionalities(40, 42, 46, 47). The ED has complex processes with its personnel having different information needs with respect to their responsibilities. Therefore, it is essential to pay attention to quality dashboard functionalities and select suitable performance indicators to be monitored by the dashboard (40). The dashboard development challenges included data sources and data quality, integration with other systems, adaptability of dashboard functionalities to user needs, and selection of appropriate performance indicators. These findings are consistent with those of Ghazisaeidi et al. (48). The main challenge in integrating dashboards with other systems is mostly related to the information technology infrastructure in the organization, which focuses on data collection through different data sources, data integration, and their linkage to the dashboard through the most appropriate method. (49) Designing a suitable architecture for supporting the dashboard requires identification of different data hosting structures, various methods of data replication and transfer, and the best query language for this data structure (50). Moreover, the identification of data sources—the processes used for data generation— and precise, comprehensive, and reliable datasets for generating high-quality data are major topics in dashboard development, which increase ED users’ trust in dashboard information (48). Research findings also demonstrate that continuous implementation with small changes can greatly contribute to the success of dashboard in meeting users’ needs(48, 50).

**Table 1:** Search formula

Search formula	Terms	No.
**“dashboard” OR “electronic whiteboard” OR “emergency department dashboard” OR “emergency department information system” OR “status board” OR “electronic tracking board”**	**Dashboard**	**1**
**“emergency department” OR “emergency” OR “emergency medicine” OR “emergency medical services” OR “emergency unit”**	**ED**	**2**
**1 and 2**		**3**

ED: emergency department.

**Table 2:** Search strategy

**Database**	**Search ** **terms**
**PubMed**	(Emergency department dashboard[TIAB] OR whiteboard[TIAB] OR emergency department information system[TIAB] OR status board[TIAB] OR Electronic tracking board[TIAB] OR dashboard[TIAB]) AND (emergency department[MeSH] OR emergency department[TIAB] OR emergency[TIAB] OR emergency medicine[TIAB] OR Emergency Medical Services[MeSH] OR emergency unit[TIAB]) NOT review
**Web of Science**	TS=(dashboard OR "electronic whiteboard" OR “Emergency department dashboard” OR “emergency department information system” OR "status board" OR "Electronic tracking board") AND TS=("emergency department" OR emergency OR "emergency medicine" OR "Emergency Medical Services" OR "emergency unit")
**Embase**	('emergency department dashboard':ab,ti OR whiteboard:ab,ti OR 'emergency department information system':ab,ti OR dashboard:ab,ti OR 'electronic tracking board':ab,ti OR 'status board':ab,ti) AND ('emergency ward'/exp/mj OR 'emergency medicine'/exp/mj OR 'emergency health service'/exp/mj OR 'emergency ward':ab,ti OR 'emergency medicine':ab,ti OR 'emergency health service':ab,ti)
**Scopus**	((TITLE-ABS (*dashboard*) OR TITLE-ABS ( *"electronic whiteboard*" ) OR TITLE-ABS ( "*Emergency department dashboard*" ) OR TITLE-ABS ( "*emergency department information system*" ) OR TITLE-ABS ( "*status board*" ) OR TITLE-ABS ( "*Electronic tracking board*" ) ) AND ( TITLE-ABS ( "*emergency department*" ) OR TITLE-ABS ( *emergency* ) OR TITLE-ABS ( "*emergency medicine*" ) OR TITLE-ABS ( "*Emergency Medical Services*" ) OR TITLE-ABS ( "*emergency unit*" ) ) )

**Table 3:** Evaluated key performance indicators of emergency department in included articles

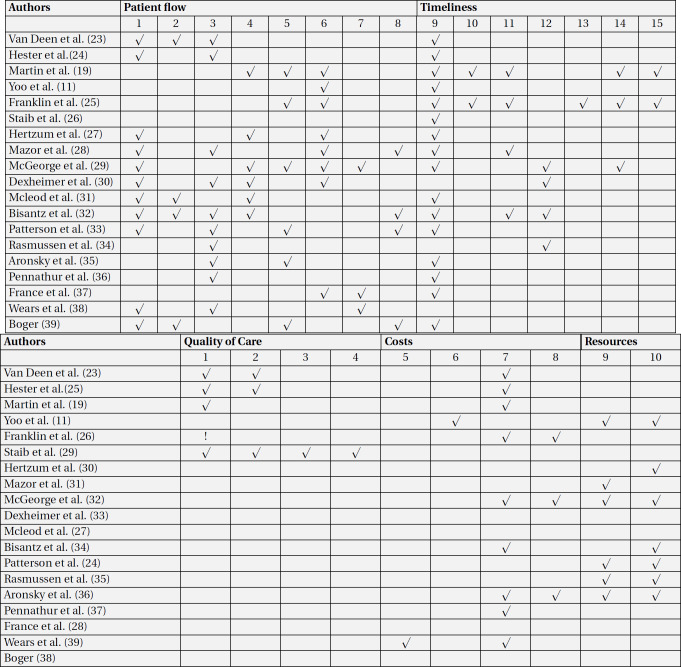

1. The total number of people visiting the ED

2. How patients visit the ED

3. The number of patients discharged, and the type of discharge

4. The number of patients based on the triage level

5. The number of patients leaving the ED without primary evaluation and treatment

6. The number of patients in the ED

7. The number of patients waiting to be visited

8. The number of patients discharged from the ED in six hours

9. The patients’ mean length of stay

10. The mean time elapsed since the doctor’s request for admission until the assignment of a room/bed to the patient in the inpatient ward

11. The time of triaging (per each triage level)

12. The mean time elapsed since the patients’ arrival in the ED until the onset/beginning of triaging

13. The mean time elapsed since the patients’ arrival in the ED until admission at the ward

14. The mean time elapsed since the patients’ arrival in the ED until doctor's visit

15. The mean time elapsed since the orders are recorded until the results (tests, imaging, electrocardiography) are ready.

**Table 4:** Specifications of the dashboards examined in the papers

**Author**	**quality dashboard functionalities**	**Challenges**
Van Deen et al. (23) 2019; USA	Reporting Drill-down and drill-up Drill-through Customization	The data entered into the dashboard were not reliable or sufficiently accurate.
Hester et al.(25) 2019;Denmark	Reporting Real-time information display	The designed dashboard functionalities were not compatible with the work processes of the healthcare providers in the ED.
Martin et al. (19) 2018; USA	Resource management Reporting Real-time information display Customization	N/A
Yoo et al. (11) 2018; South Korea	Real-time information displayAutomated data collection Reporting Alert Customization	The designed dashboard was evaluated only in one ED, and it might not be useful because of the hospital’s different specialties and the use of different performance indicators.
Franklin et al. (26) 2017; USA	Resource management Alert Reporting Real-time information display Drill-down and drill-up Drill-through	The information displayed by the dashboard did not meet the diverse needs of ED users.
Staib et al. (29) 2017; Australia	Real-time information display Reporting	N/A
Hertzum et al. (30) 2016; Denmark	Resource management Reporting Real-time information display Customization	The challenges included a lack of integration between the dashboard and the other systems at the hospital, and inputting the information manually.
Mazor et al. (31) 2016; Israel	Alert Resource management Reporting Customization Drill-down and drill-up Drill-through	The research limitation was that the evaluation was performed in a simulated environment.
McGeorge et al. (32) 2015; USA	Reporting Resource management Customization	The research limitation was that the evaluation was performed in a simulated environment.
Dexheimer et al. (33) 2013; USA	Resource management Alert Reporting Customization	A problem in interpreting the information displayed by the dashboard, and breaching confidentiality as some patient demographic information was displayed in the dashboard.
Mcleod et al. (27) 2010; Canada	Real-time information display Automated data collection Reporting	N/A
Bisantz et al. (34) 2010; USA	Resource management Reporting Customization	A problem with understanding the type of information displayed in the dashboard, and the dashboard’s lack of adaptability to work processes and the doctors’ information needs
Patterson et al. (24) 2010; USA	Automated data collection Resource management Alert Reporting Customization	Lack of integration with other hospital systems, and inputting the data manually
Rasmussen et al. (35) 2010;Denmark	Alert Reporting Customization	Lack of compatibility between the features of the new system and the work processes in the ED
Aronsky et al. (36) 2007; USA	Real-time information display Automated data collection Alert Reporting Customization Drill-down and drill-up Drill-through	Displaying information such as patient name and test results on the dashboard large screen violated patient information confidentiality.
Pennathur et al. (37) 2007; USA	Alert Reporting Customization	Lack of compatibility with workflows, especially during triaging and tracking the stages of care
France et al. (28) 2005; USA	Resource management Reporting	N/A
Wears et al. (39) 2003; USA	Alert Reporting	Low flexibility, no customization feature
Boger (38) 2003; USA	Reporting Alert	N/A

ED: emergency department.

**Table 5:** Risk of bias assessment in included studies based on effective practice and organization of care (EPOC) tools

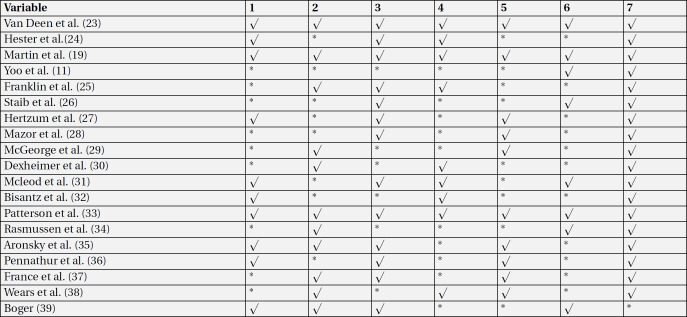

1. Intervention independent of other changes

2. Shape of the intervention effect pre-specified

3. Intervention unlikely to affect data collection

4. Knowledge of the allocated interventions adequately prevented during the study

5. Incomplete outcome data adequately

6. Selective outcome reporting

7. Other risks of bias

## 5. Limitations

There are limitations with the current study that could be addressed in future research. First of all, the current study reviewed studies published in English and there might be useful relevant studies that were excluded. In addition, although we systematically studied the key performance indicators of quality dashboard in the emergency department, we did not include studies that addressed clinical dashboards of this department due to aim of the study. In the analysis step, an attempt was made to avoid or minimize the risk of bias, as any possible discrepancy experienced by either of the first three authors were discussed in group. However, there may have been issues that did not catch our attention. 

## 6. Conclusion and suggestion

The findings of the current study indicated that applying key performance indicators for ED quality dashboard, including quality of care, patient flow, timeliness, costs, and resources, could improve utilization management and quality of care. Moreover, a quality dashboard should support functions such as customization, reporting, real-time information display, resource management, alert creation, and automated data collection. In addition, the use of drill-up, drill-down, and drill-through features could help improve efficiency and effectiveness of ED quality dashboard. With respect to the challenges noted, it is suggested that in further developments of ED dashboard, attention should be paid to data sources, the quality of data, and integration with other systems both in the ED and in other hospital departments.

## 7. Declarations:

### 7.1. Acknowledgment

 None.

### 7.2 Authors' Contributions

Concept and study design: SA, RR, HM, MVA; literature search, study selection and data collection: SA; analysis and interpretation: SA, RR, HM; Writing the article: SA; critical revision of the article: RR, HM; final approval of the article: SA, RR, HM, MVA.

### 7.3 Funding

This systematic review did not receive any funding.

### 7.4 Ethical approval

 This review was part of a larger study, approved by the Ethics Committee of Shahid Beheshti University of Medical Sciences, Iran (IR.SBMU.RETECH.REC.1399.565).

### 7.5 Conflicts of interest

 The authors declare that they have no competing interests.

## References

[1] Steptoe AP, Corel B, Sullivan AF, Camargo CA (2011). Characterizing emergency departments to improve understanding of emergency care systems. Int J Emerg Med..

[2] Higginson I (2012). Emergency department crowding. Emergency medicine journal : EMJ..

[3] Morley C, Unwin M, Peterson GM, Stankovich J, Kinsman L (2018). Emergency department crowding: A systematic review of causes, consequences and solutions. PLoS One..

[4] Taylor TB (2004). Information management in the emergency department. Emerg Med Clin N Am..

[5] Ahanhanzo YG, Kpozehouen A, Sopoh G, Sossa-Jérôme C, Ouedraogo L, Wilmet-Dramaix M (2016). Management of information within emergencies departments in developing countries: analysis at the National Emergency Department in Benin. Pan Afr Med J..

[6] Selck FW, Decker SL (2016). Health Information Technology Adoption in the Emergency Department. Health Serv Res..

[7] Callen J, Paoloni R, Li J, Stewart M, Gibson K, Georgiou A (2013). Perceptions of the effect of information and communication technology on the quality of care delivered in emergency departments: a cross-site qualitative study. Ann Emerg Med..

[8] Handel DA, Wears RL, Nathanson LA, Pines JM (2011). Using information technology to improve the quality and safety of emergency care. Academic emergency medicine : official journal of the Society for Academic Emergency Medicine..

[9] Fairbanks RJ, Guarrera TK, Karn KS, Caplan SH, Shah MN, Wears RL (2008). Interface Design Characteristics of a Popular Emergency Department Information System. Proc Hum Factors Ergon Soc Annu Meet..

[10] Wilbanks BA, Langford PA (2014). A review of dashboards for data analytics in nursing. Computers, informatics, nursing : CIN..

[11] Yoo J, Jung KY, Kim T, Lee T, Hwang SY, Yoon H (2018). A real-time autonomous dashboard for the emergency department: 5-year case study. JMIR mHealth uHealth.

[12] Pauwels K, Ambler T, Clark BH, LaPointe P, Reibstein D, Skiera B (2009). Dashboards as a Service: Why, What, How, and What Research Is Needed?. J Serv Res..

[13] Carroll C, Flucke N, Barton AJ (2013). The use of dashboards to monitor quality of care. Clinical nurse specialist CNS..

[14] Randell R, Alvarado N, McVey L, Ruddle RA, Doherty P, Gale C (2020). Requirements for a quality dashboard: Lessons from National Clinical Audits. AMIA Annu Symp Proc..

[15] Park KW, Smaltz D, McFadden D, Souba W (2010). The operating room dashboard. The Journal of surgical research..

[16] Karami M, Safdari R (2016). From Information Management to Information Visualization. Appl Clin Inform..

[17] Russell M, Hogg M, Leach S, Penman M, Friel S (2014). Developing a general ward nursing dashboard. Nursing standard (Royal College of Nursing (Great Britain) : 1987)..

[18] Koch SH, Weir C, Westenskow D, Gondan M, Agutter J, Haar M (2013). Evaluation of the effect of information integration in displays for ICU nurses on situation awareness and task completion time: A prospective randomized controlled study. Int J Med Inform..

[19] Martin N, Bergs J, Eerdekens D, Depaire B, Verelst S (2018). Developing an emergency department crowding dashboard: A design science approach. Int Emerg Nurs..

[20] Moher D, Liberati A, Tetzlaff J, Altman DG (2009). Preferred reporting items for systematic reviews and meta-analyses: the PRISMA statement. PLoS medicine..

[21] Cochrane Effective Practice and Organisation of Care (EPOC) Suggested risk of bias criteria for EPOC reviews. EPOC Resources for review authors,2017. Cited[20 September 2020].

[22] Sørup CM, Jacobsen P, Forberg JL (2013). Evaluation of emergency department performance - a systematic review on recommended performance and quality-in-care measures. Scand J Trauma Resusc Emerg Med..

[23] van Deen WK, Cho ES, Pustolski K, Wixon D, Lamb S, Valente TW (2019). Involving end-users in the design of an audit and feedback intervention in the emergency department setting–a mixed methods study. BMC health services research..

[24] Patterson ES, Rogers ML, Tomolo AM, Wears RL, Tsevat J (2010). Comparison of extent of use, information accuracy, and functions for manual and electronic patient status boards. Int J Med Inform..

[25] Hester G, Lang T, Madsen L, Tambyraja R, Zenker P (2019). Timely data for targeted quality improvement interventions: use of a visual analytics dashboard for bronchiolitis. Appl Clin Inform..

[26] Franklin A, Gantela S, Shifarraw S, Johnson TR, Robinson DJ, King BR (2017). Dashboard visualizations: Supporting real-time throughput decision-making. Journal of biomedical informatics..

[27] McLeod B, Zaver F, Avery C, Martin DP, Wang D, Jessen K (2010). Matching capacity to demand: a regional dashboard reduces ambulance avoidance and improves accessibility of receiving hospitals. Academic emergency medicine : official journal of the Society for Academic Emergency Medicine..

[28] France DJ, Levin S, Hemphill R, Chen K, Rickard D, Makowski R (2005). Emergency physicians’ behaviors and workload in the presence of an electronic whiteboard. International journal of medical informatics..

[29] Staib A, Sullivan C, Jones M, Griffin B, Bell A, Scott I (2017). The ED-inpatient dashboard: Uniting emergency and inpatient clinicians to improve the efficiency and quality of care for patients requiring emergency admission to hospital. Emergency medicine Australasia : EMA..

[30] Hertzum M, Simonsen J (2016). Effects of electronic emergency-department whiteboards on clinicians’ time distribution and mental workload. Health informatics journal..

[31] Mazor I, Heart T, Even A (2016). Simulating the impact of an online digital dashboard in emergency departments on patients length of stay. Journal of Decision Systems..

[32] McGeorge N, Hegde S, Berg RL, Guarrera-Schick TK, LaVergne DT, Casucci SN (2015). Assessment of Innovative Emergency Department Information Displays in a Clinical Simulation Center. J Cogn Eng Decis Mak..

[33] Dexheimer JW, Kennebeck S (2013). Modifications and integration of the electronic tracking board in a pediatric emergency department. Pediatric emergency care..

[34] Bisantz A, Pennathur P, Guarrera T, Fairbanks R, Perry S, Zwemer F (2010). Emergency Department Status Boards: A Case Study in Information Systems Transition. J Cogn Eng Decis Mak..

[35] Rasmussen R, Fleron B, Hertzum M, Simonsen J, editors (2010). Balancing tradition and transcendence in the implementation of emergency-department electronic whiteboards. Selected papers of the information systems research seminar in Scandinavia.

[36] Aronsky D, Jones I, Lanaghan K, Slovis CM (2008). Supporting patient care in the emergency department with a computerized whiteboard system. J Am Med Inform Assoc..

[37] Pennathur PR, Bisantz AM, Fairbanks RJ, Perry SJ, Zwemer F, Wears RL, editors Assessing the impact of computerization on work practice: Information technology in emergency departments. Proceedings of the Human Factors and Ergonomics Society Annual Meeting.

[38] Boger E (2003). Electronic tracking board reduces ED patient length of stay at Indiana Hospital. J Emerg Nurs..

[39] Wears RL, Perry SJ, Shapiro M, Beach C, Croskerry P, Behara R, editors (2003). A comparison of manual and electronic status boards in the emergency department: what's gained and what's lost? Proceedings of the Human Factors and Ergonomics Society Annual Meeting.

[40] Randell R, Greenhalgh J, Wyatt J, Gardner P, Pearman A, Honey S (2015). Electronic whiteboards: review of the literature. Studies in health technology and informatics..

[41] Dowding D, Randell R, Gardner P, Fitzpatrick G, Dykes P, Favela J (2015). Dashboards for improving patient care: review of the literature. Int J Med Inform..

[42] Janssen A, Donnelly C, Kay J, Thiem P, Saavedra A, Pathmanathan N (2020). Developing an Intranet-Based Lymphedema Dashboard for Breast Cancer Multidisciplinary Teams: Design Research Study. J Med Internet Res..

[43] Institute of Medicine (US) Committee on Quality of Health Care in America (2001). Crossing the Quality Chasm: A New Health System for the 21st Century.

[44] Diley I, Badrinath P, Annon S (2014). Is mortality a good indicator of the clinical quality of National Health Service hospitals? A cross-sectional study of outlier trusts for mortality indices using quality dashboards. JRSM open..

[45] Ngantcha M, Le-Pogam M-A, Calmus S, Grenier C, Evrard I, Lamarche-Vadel A (2017). Hospital quality measures: are process indicators associated with hospital standardized mortality ratios in French acute care hospitals?. BMC health services research..

[46] Eckerson WW (2010). Performance dashboards: measuring, monitoring, and managing your business.

[47] Jeffs L, Beswick S, Lo J, Lai Y, Chhun A, Campbell H (2014). Insights from staff nurses and managers on unit-specific nursing performance dashboards: a qualitative study. BMJ quality & safety..

[48] Ghazisaeidi M, Safdari R, Torabi M, Mirzaee M, Farzi J, Goodini A (2015). Development of performance dashboards in healthcare sector: key practical issues. Acta Informatica Medica..

[49] Minnigh TR, Gallet J (2009). Maintaining quality control using a radiological digital X-ray dashboard. J Digit Imaging..

[50] Rasmussen NH, Bansal M, Chen CY (2009). Business dashboards: a visual catalog for design and deployment.

